# Probing the Hypersalience Hypothesis—An Adapted Judge-Advisor System Tested in Individuals With Psychotic-Like Experiences

**DOI:** 10.3389/fpsyt.2021.612810

**Published:** 2021-03-04

**Authors:** Jakob Scheunemann, Rabea Fischer, Steffen Moritz

**Affiliations:** Department of Psychiatry and Psychotherapy, University Medical Center Hamburg-Eppendorf, Hamburg, Germany

**Keywords:** schizophrenia, psychosis, cognitive biases, jumping to conclusions, belief flexibility, information processing, decision-making, psychosis-prone

## Abstract

Individuals with psychotic-like experiences and psychosis gather and use information differently than controls; in particular they seek and rely on less information or over-weight currently available information. A new paradigm, the judge-advisor system, has previously been used to investigate these processes. Results showed that psychosis-prone individuals tend to seek less advice but at the same time use the available advice more. Some theoretical models, like the hypersalience of evidence-matching hypothesis, predict that psychosis-prone individuals weight recently available information to a greater extent and thus provide an explanation for increased advice-weighting scores in psychosis-prone individuals. To test this model, we adapted the previously used judge-advisor system by letting participants receive consecutively multiple pieces of advice. To meet this aim, we recruited a large MTurk community sample (*N* = 1,396), which we split in a group with high levels of psychotic-like experiences (at least 2 *SD* above the mean, *n* = 80) and a group with low levels of psychotic-like experiences (maximum 0.5 *SD* above the mean, *n* = 1,107), using the Community Assessment of Psychic Experiences' positive subscale. First, participants estimated five people's age based on photographs. Then, they received consecutive advice in the form of manipulated age estimates by allegedly previous participants, with outliers in some trials. After each advice, participants could adjust their estimate. This procedure allowed us to investigate how participants weighted each currently presented advice. In addition to being more confident in their final estimates and in line with our preregistered hypothesis, participants with more frequent psychotic-like experiences did weight currently available advice more than participants with less frequent psychotic-like experiences. This effect was especially pronounced in response to outliers, as fine-grained *post-hoc* analysis suggested. Result thus support models predicting an overcorrection in response to new incoming information and challenges an assumed general belief inflexibility in people with psychotic experiences.

## Introduction

Several cognitive models suggest biases in information processing as a factor for the formation and maintenance of psychosis (1, 2). Prominent biases are the jumping to conclusions bias [JTC; (3)], bias against disconformitory evidence [BADE; (4)], and overconfidence (5). In the best-known paradigm to tap data gathering, the beads task (6) and its variants the fish-task (7) and box task (8), participants collect information before making a decision on a probabilistic reasoning task. Yet, the task faces a number of caveats including low reliability and comprehensibility. In this study, we used an alternative paradigm, an adaptation of the judge-advisor system, to tap different biases concurrently [JAS; (9)]. The JAS has been developed in the field social and organizational psychology [for a review, see Bonaccio and Dalal (10)], but has recently been applied in clinical research (11, 12). Our adaptation allows to investigate how participants seek and use advice on estimation tasks, which are processes that likely involve the cognitive biases belief flexibility, jumping to conclusions, and confidence in judgements.

In the JAS task, a participant makes an initial estimate (e.g., in this study about a person's age based on a photograph) and then receives advice [e.g., in this study (fabricated) answers by previous participants]. After a participant received advice, they can adjust their estimate. Kaliuzhna et al. (12) used the JAS in a study on patients with schizophrenia: In the first part, participants made estimates on knowledge questions (e.g., when was UNO created?). In the second part, participants received estimates by another individual as advice and had the option to adjust it. Against the initial hypothesis, patients with schizophrenia adjusted their estimate more than healthy controls in response to advice. Likewise, in one of our previous studies on participants along the psychosis-spectrum, participants with more frequent psychotic-like experiences (PLEs) did weight the first advice more than participants with less frequent PLEs (13).

The finding that individuals with more frequent psychotic(-like) experiences weighted advice more than controls is surprising considering a series of previous studies suggesting the opposite: Psychotic patients have shown to be immune toward conflicting evidence against their delusion (14). Also on delusion neutral material—for example measured with the BADE paradigm (15)—patients with schizophrenia (16) and people with more frequent PLEs (17) show a tendency to stick to initial explanations even after being confronted with evidence speaking against it. However, the hypersalience of evidence-matching hypothesis (18) provides a rationale for an increased advice weighting: According to this theory, patients with schizophrenia put more weight toward currently available information in the direction of this evidence; at the same time, previous information is considered less. If so, individuals with more frequent PLEs should weight currently available advice more than individuals with less frequent PLEs.

This hypersalience of evidence-matching hypothesis is derived from the observation of “overcorrection” in the fish task [(7); a variant of the classical beads task; (6)] intended to capture jumping to conclusions: When participants have to deduce from which of two lakes with opposite ratios of colored fish (e.g., lake 1: 80% red fish and 20% green fish; lake 2: 20% red fish and 80% green fish) a fisherman catches fish, contrary evidence—for example a green fish after three consecutive red fish—leads to an increase in probability ratings for the lake containing more green fish (but not a decreased rating for the lake containing more red fish) in schizophrenia patients compared to controls. This “overcorrection” has been observed already in one of the first studies with this task (19), and was replicated multiple times (20–22). In their analysis, Speechley et al. (18) showed that this overcorrection only applies to probability ratings to the lake favored by the current fish [match between hypothesis (lake) and information (fish)], while the probability rating of the opposing lake is not overcorrected (non-match between hypothesis [lake] and information [fish]); thus the name “hypersalience of evidence-matching hypothesis.”

### The Aberrant JAS

In the version of the JAS we are using, the participant makes an initial estimate about a person's age based on a photograph and then receives advice in the form of (fabricated) answers by previous participants. To explicitly test the hypersalience model for advice weighting, we made the following adaptations to the original JAS-paradigm: Participants received multiple, consecutive pieces of advice. Important to note is that participants did neither know nor could they influence the number of pieces of advice they would see in any given trial. However, after each advice, participants could revise their estimate. Further, we manipulated the advice so that some pieces of advice were “outliers” differing largely from the other advice (hence the name *Aberrant JAS*). The hypersalience of evidence-matching hypothesis predicts that individuals with more frequent PLEs weight these outliers stronger as they consider previous information less.

To simulate information seeking, we additionally asked participants after each advice whether they preferred more advice (which did not alter the probability whether more advice would be shown). After the final estimate, participants rated how confident they are in their decision, to investigate a possible overconfidence (5). Thus, the Aberrant JAS provides measures on the integration of consecutively incoming information, information seeking and confidence in judgements. Additionally, as has been done before by Hofheinz et al. (11) in a depression sample, we investigated the role of self-esteem in exploratory fashion, as lower self-esteem seems to be related to more advice taking (23).

### Aims and Hypotheses

This study followed two main aims. First, we wanted to test the hypothesis that individuals with more frequent PLEs weight currently available advice more than controls. Second, we wanted to optimize the JAS-paradigm for the research of mechanisms on information processing in relation to psychosis. Therefore, we designed and analyzed different sequences of advice. We performed this study on a community sample by dividing participants in groups based on the positive subscale of the Community Assessment of Psychic Experiences (24) as previously done by multiple research groups (13, 25–27). This approach has the advantage to first validate this new task on a non-burdened population, which is also free from confounds like medication that influence information processing (28, 29). Along the hypothesis that (1) participants with more frequent PLEs would weight currently available information more than participants with less frequent PLEs, we also tested the hypotheses that (2) they would weight all advice (averaged) more, (3) prefer to see less advice, (4) and are more confident in their final estimate (rated after estimate). Finally we hypothesized that (5) confidence correlates with subjective competence in task performance (rated before experiment started)—moderated by group—as previous studies suggest that patients with schizophrenia mostly feel overconfident in areas they feel competent in (30, 31).

## Methods

### Preregistration and Ethics

Before data collection (July 4th, 2019; time-stamped), we publicly preregistered the study on *As Predicted* (#21768). Our local ethics committee approved the study (#LPEK-0074)^1^.

### Recruitment

We recruited participants via Amazon Mechanical Turk (MTurk). To ensure data quality we followed suggestions by Kees et al. (32) which means that participants could only participate if they had a U.S. IP address, had an acceptance rate of 95% or higher based on at least 100 previous MTurk tasks (so called human intelligence tasks), and had not participated in a previous study by our working group before.

Of 1,616 people who had begun the survey, 1,570 finished. In line with our preregistered protocol, we excluded (blind to results) 71 participants because of poor results on an attention assessment (self-rated attentiveness during the study of ≤ 5 on a 7-point Likert scale), 75 participants due to an implicit attention test [item within the sociodemographic questionnaire: “People vary in the amount they pay attention to these kinds of surveys. Some take them seriously and read each question, whereas others go very quickly and barely read the questions at all. If you have read this question carefully, please write the word yes in the blank box below labeled other. There is no need for you to respond to the scale below” (33)] and 28 due to excessive speeding as defined by a response time of 50% of the median completion time (cut off: 4.99 min). One participant was excluded due to an error by the user (for more details, see section Preprocessing below). After removing 176 (11.2%) participants in total, 1,395 participants were included in the analysis.

## Materials

### Community Assessment of Psychic Experience Scale (CAPE)

We asked all participants to fill out the 20-item long positive subscale of the Community Assessment of Psychic Experience Scale (24), measuring positive psychotic-like experience. Items are answered on a scale from 1 (“never”) to 4 (“nearly always”). The internal consistency in our sample (Cronbach's α = 0.898) was similar to previous studies [meta-analytic mean α = 0.91; (34)]. As defined in the preregistration report, the sample was divided into PLE-High with participants scoring at least two standard deviations above the mean (*n*_*PLEs*−*High*_ = 80) and PLE-Low (*n*_*PLEs*−*Low*_ = 1,106) with participants scoring at maximum 0.5 standard deviations above the mean. This approach has been used in multiple psychometric high-risk studies (13, 25–27). We also report results of the eight item long depressive subscale. The negative subscale was not assessed.

### Rosenberg Self-Esteem Scale (RSES)

Rosenberg's Self-Esteem Scale (35) is a 10-item long self-report inventory measuring global self-esteem answered on a four-point Likert scale from 1 (“strongly disagree”) to 4 (“strongly agree”).

### Subjective Competence

Before the estimation task started, participants responded once to the question “How good do you judge yourself to be at estimating other people's age?” using a Likert scale from 1 (“very good”) to 5 (“very bad”).

### The Aberrant Judge-Advisor System

The sequence of the experiment was adopted from the classical judge-advisor systems [JAS; (10)]: That means participants first made an initial judgement, then they received advice along with the option to adjust their initial judgement. The most relevant outcome is whether and how much participants adjusted their initial judgement in response to the advice (or in this case to the sequence of pieces of advice).

**Figure 1:**
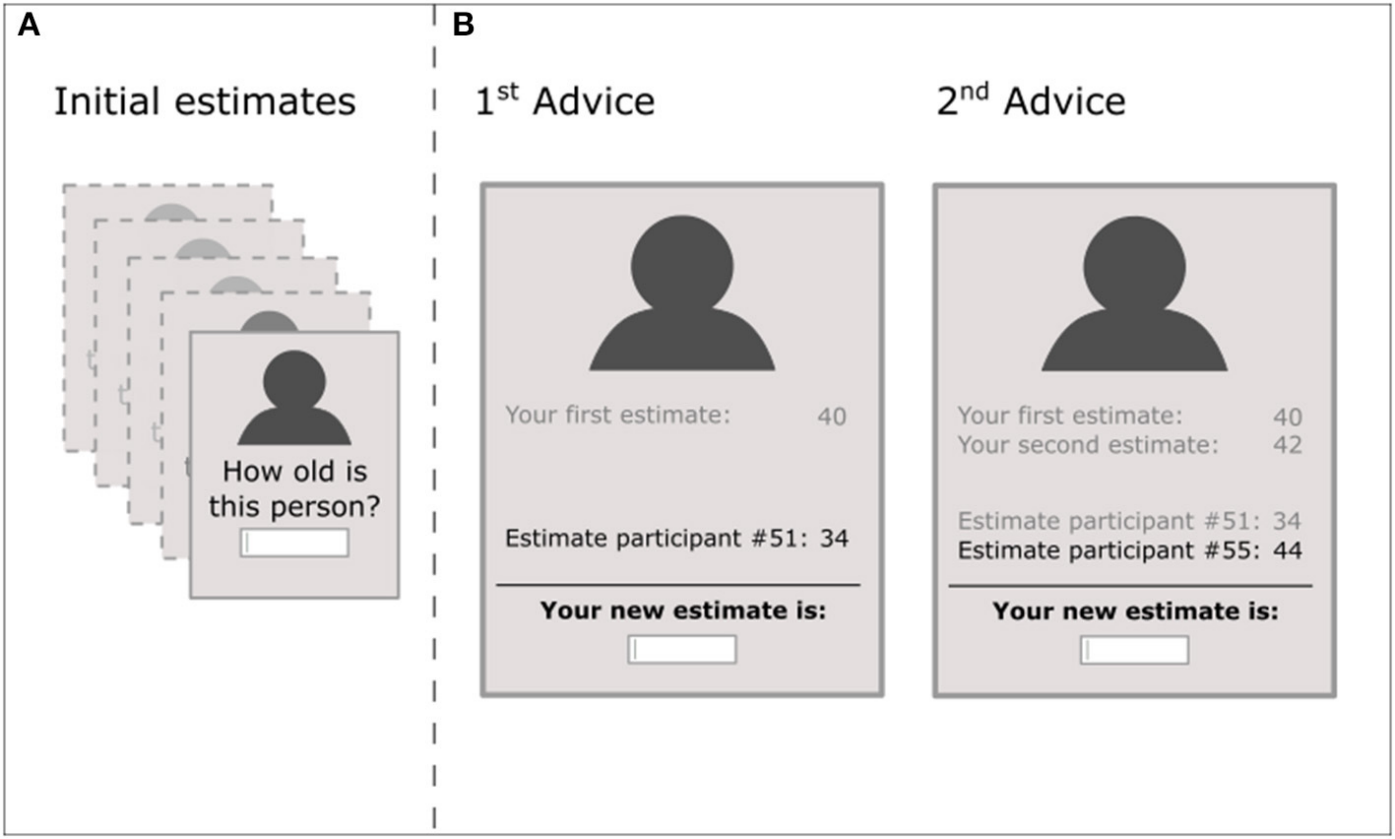
Schematic representation of the Aberrant JAS. **(A)** Participants consecutively saw portrait pictures of five different persons, for which they gave each an initial estimate. **(B)** In the second step, participants saw the same portrait pictures in the same order along with advice, which we framed in the experiment as “estimates from previous participants.” As an example, we illustrated trial 3: The first advice was −15% to the initial estimate and participants made a new estimate. Thereafter, participants saw the second advice (+10% to the initial estimate) and again gave a new estimate. This estimate was the final estimate, as the trial ended after two pieces of advice (number of pieces of advice per trial were unknown to participants and ranged from 1 to 5). As illustrated, in all steps, participants saw the portrait picture, all their previous estimates, all previous advice and the new advice highlighted. Participants gave their estimates by typing in a number in digits for which there was no time constraint. As additional measures (not depicted), participants responded after each new estimate whether they would prefer to see more estimates from previous participants (along the information that this does not influence the number of pieces of advice). After each trial terminated, participants rated their confidence in their last/final estimate.

Figure 1 illustrates the adapted Aberrant Judge-Advisor System: At the start, participants saw five portraits (770 × 512 pixels) with individuals of White race of various ages (3 men and 2 women) taken from the Siblings Database of the CG&V Group (36). Each picture was presented one at a time and participants first gave an initial estimate on the age of the person in the picture (by typing the age in digits). Only after participants have given all five initial estimates, we informed participants that they would see the same pictures again along with “randomly drawn estimates from participants, who gave those estimates in a previous small study with 100 participants.” These answers were in fact pre-determined and functioned as advice (we avoided the term “advice” in the instructions). For each picture, the total number of pieces of advice varied, but in each case, the pieces of advice were revealed one at a time. Participants did not have any knowledge about the total number of pieces of advice. Table 1 depicts the exact number and distances of advice for all five trials, along with explanations of the intended rationale. Important are the “outliers” in trial 2 and 4, which we defined as advice that deviated largely from previous, little divergent, advice. The order of the five trials was fixed for all participants, the corresponding picture, however, was randomly allocated to the trials. All advice was presented along the picture, all previous pieces of advice on this picture and all previous estimates the participant made on this picture. For each presented advice, participants gave a new, possibly revised estimate. To do so, they had to type in their estimate in digits again.

**Table 1:** Explanation of trials—relative distance of advice to the initial estimate and the intended rationale.

**Rule**			
**Trial 1**			
	1st Advice	−7.5%	The purpose of this trial was to ensure participants believe every estimate might be the last one
**Trial 2**			
	1st Advice	+17.5%	Note the outlier of the 4th advice
	2nd Advice	+30%	
	3rd Advice	+25%	
	4th Advice	−10%	
**Trial 3**			
	1st Advice	−15%	In this trial, there are two opposing/contradicting pieces of advice
	2nd Advice	+10%	
**Trial 4**			
	1st Advice	−5%	Note the outlier of the 4th advice, after all previous pieces of advice were confirmatory of the initial estimate
	2nd Advice	+2.5%	
	3rd Advice	+0%	
	4th Advice	+22.5%	
**Trial 5**			
	1st Advice	+12%	All advice hinted in the same direction
	2nd Advice	+14%	
	3rd Advice	+8%	
	4th Advice	+18.5%	
	5th Advice	+10%	

Further, they answered the question “Would you prefer to see more estimates from others before making a final decision?” The answer to this question, however, would not influence the number of pieces of advice. Participants knew that their answer would have no influence as we have pointed this out: “(Your answer does not influence whether you see more estimates or not).” After participants had seen the predetermined number of pieces of advice, the estimate was set as final. Participants subsequently rated their confidence. There was no time constraint during any part of the experiment.

### Scoring

#### Preprocessing

Of 24,927 estimates made, 5 estimates were outside the range of 20–83 and deleted because they likely represented typos (age estimates were 4, 391, 412, 445, and 569). One participant gave this answer at the first estimate. Consequently, the resulting generated advice was also unrealistic; hence, we excluded this participant from the analysis as this revealed to the participant that the advice was not real, but automatically generated.

#### Information Integration: Advice Weighting

The most common procedure to calculate the degree a participant integrates advice is relative advice weighting (RAW). The basic formula is (RAW = [final estimate—initial estimate]/[advice—initial estimate]; formula 1), which is the ratio between the change in estimate by the distance of advice. We adapted the formula for relative current advice weighting and relative average advice weighting.

*Relative current advice weighting* (RCAW) aims at capturing the weight a participant put toward the advice presented last. This is the most relevant outcome regarding our hypotheses. We were interested, for example, in how much a participant weighted the fourth advice for the second picture. We a priori defined relative current advice weighting as the change in estimates between pieces of advice, relative to the distance between the new/current advice and the previous estimate (RCAW = [new estimate—previous estimate]/[current advice−previous estimate]; formula 2). This formula has an intuitive interpretation: If a participant does not change the estimate, the RCAW is 0. If the participant follows the advice completely, the score will be 1. If the participant takes the middle between the previous estimate and the new advice, the RCAW will be 0.5. A negative score would mean that the participant changed their estimate in the opposite direction to the advice (e. g., in trial 1: initial estimate: 40, advice: 37, new estimate: 42; would results in RCAW = −0.67). No score was calculated for the case that the current advice equaled the previous estimate, as the denominator of this formula 2 resulted in zero in this case.

*Relative average advice weighting* (RAAW) uses the basic formula 1 described above, but averages the pieces of advice, as previously done [e.g., (37)]. The resulting formula is RAAW = [final estimate—initial estimate]/[mean advice—initial estimate] (formula 3). For illustration, take picture 5 (also, see Table 1): If a participant gave an initial estimate of 30, the five pieces of advice (34, 34, 32, 36, and 33) averaged to 33.8. Hence, an adjustment by 1 year from 30 to 31 led to RAAW = (31–30)/(33.8–30) = 1/3.8 ≈ 0.26; equivalent to saying the advice was integrated by 26%.

It is important to note that for both RCAW and RAAW, scores outside the range 0–1 are not unlikely. In picture 4, for example, the average advice is 5% above the initial estimate. With an initial estimate of 25, the average advice was 26.5. Yet, the outlier at the final estimate (advice: 31 years) might have changed the estimate to 27, resulting in a RAAW score of 2/1.5 = 1.33.

#### Information Sampling: Number of Preferred Advice (NoPA)

After each piece of advice, we asked participants “Would you prefer to see more estimates from others before making a final decision?” Hereby “Yes, I would prefer more estimates” was scored as 1 and “No, I have enough information” was scored as 0. We informed participants that the answer does not influence the number of pieces of advice shown. We calculated a mean Number of Preferred Advice (NoPA) score per trial, which ranged between 0 and 1 and represents the percentage participants on average preferred to see more advice per trial. If, for example in trials 4 (see, Table 1) a particiapnt prefered to see more advice after the first and fourth advice (each scored as 1), but not after the second and third advice (each scored as 0) the NoPA score in this trial was 2/4 = 0.5.

#### Confidence

At the end of each trial, participants rated their confidence in their estimate (“Please indicate how confident you are in your estimate:”) on a 4-point scale from 1 (“not very confident”), 2 (“moderately confident”), 3 (“very confident”) to 4 (“100% confident”). Note that the confidence score differs to subjective competence described above: Subjective competence is rated before the task and assesses the general competence in this type of task (in this case the competence in estimating people's age based on a picture), while confidence refers to the confidence after each trial has ended.

### Procedure and Preregistered Analysis

After participants gave informed consent, they answered the CAPE, RSE, and rated their subjective competence in estimating ages. Then, they completed the JAS. Finally, they rated their subjective competence again and provided sociodemographic information.

We computed all main analyses as indicated in the public AsPredicted protocol. We tested the a priori hypothesis of increased RCAW scores, RAAW scores and confidence as well as well as the lower preference to see more advice (NoPA) for the PLEs-High group compared to the PLEs-Low group with Welch's *t*-tests. For this, scores were averaged on the subject level across trials; for the main analysis of RCAW, we additionally averaged this score across advice per trial. Further, we calculated a correlation between Subjective Competence (rated before the task) and Confidence (rated at the end of each trial) and tested whether this correlation was moderated by group. Subjective competence and group were centered for this moderation analysis. We tested whether correlations are significantly different from zero with Student's *t*-tests. The role of self-esteem was analyzed in exploratory fashion.

## Results

Sociodemographic and core psychopathological data is summarized in Table 2, self-reported lifetime diagnoses in Supplementary Table 1. Participants in PLEs-High reported (non-significantly) more psychiatric diagnoses, were more frequently male, younger, less educated, and racially more diverse than participants in PLEs-Low.

**Table 2:** Participants' characteristics and group differences.

	**PLEs-High**	**PLEs-Low**	**Group differences**	
	**(*n* = 80)**	**(*n* = 1,106)**		
**Gender**			χ^2^ (2) = 15.591	*p <* 0.001
Female	36.25%	58.86%		
Male	62.50%	40.69%		
A different term than female or male/I wish not to answer^a^	0.00%	0.45%		
Age	33.69 (*SD =* 10.80; *range* = [19, 63])	39.41 (S*D =* 12.81; *range* = [18, 88])	*t*(98.83) = 4.513	*p <* 0.001
**Level of Education**			χ^2^ (6) = 7.088	*p =* 0.313
Less than high school	1.25%	0.45%		
High school graduate	12.50%	10.49%		
Some college	25.00%	23.96%		
2-year degree	5.00%	11.66%		
4-year degree	45.00%	37.97%		
Professional degree	11.25%	13.38%		
Doctorate	0.00%	2.08%		
Years of Education	12.80 (*SD =* 5.14)	14.90 (*SD =* 5.10)	*t*(90.601) = 3.522	*p <* 0.001
**Race/Ethnicity**^**b**^			χ^2^ (6) = 17.637	*p <* 0.007
American Indian or Alaska Native	2.50%	0.72%		
Asian or Asian American	8.75%	6.78%		
Black or African American	17.50%	7.23%		
Latino or Hispanic	10.00%	5.70%		
Native Hawaiian or Pacific Islander	0.00%	0.09%		
White or European American	65.00%	80.92%		
Neither/A term not listed above/I wish not to answer^a^	1.25%	1.63%		
**Psychopathology**				
CAPE-positive	2.467 (*SD =* 0.282)	1.289 (*SD =* 0.163)	*t*(82.846) = 36.852	*p <* 0.001
CAPE-depression	2.489 (*SD =* 0.566)	1.821 (*SD =* 0.434)	*t*(85.856) = 10.344	*p <* 0.001
Rosenberg Self-Esteem Scale	3.001 (*SD =* 0.588)	2.660 (*SD =* 0.616)	*t*(91.966) = 4.988	*p <* 0.001

a Answers were summarized;

b *multiple answers were possible*.

### Preregistered Analysis

There were no major deviations from the preregistered protocol. First, the number of participants completed the study were 1,570 instead of 1,500 unintendedly caused by wrong settings on MTurk. Even though not specified in the preregistered protocol, we report Cohen's effect size parameter *d* along with the preregistered statistical tests, including its 95% confidence interval (95% CI). For the formula 2 RCAW, we also did not specify the scenario in which the new advice equaled the previous estimate: The denominator for the formula RCAW would then be zero, thus no score was calculated (see above in Methods section).

As predicted, PLE-High weighted current advice (RCAW) more than PLE-Low with a medium effect size (RCAW PLEs-High: 0.20 (*SD* = 0.28), RCAW PLEs-Low: 0.13 (*SD* = 0.13); (*t*(81.226) = 2.380); *p* = 0.020; *d* = 0.54, 95% CI [0.31, 0.77]). This means, participants from the PLEs-High group adjusted their estimates more in response to new information available than participants from the PLEs-Low group. For the averaged advice (RAAW), group differences were in the expected direction, but the group difference was not significant (RAAW PLEs-High: 0.45 (*SD* = 0.73), RAAW PLEs-Low: 0.36 (*SD* = 0.42); (*t*(82.945) = 1.076); *p* = 0.285; *d* = 0.20, 95% CI [−0.03, 0.42]). Against our hypothesis, PLE-High preferred to see more advice, even though this difference was not significant (NoPA PLEs-High: 0.68 (*SD* = 0.92), NoPA PLEs-Low: 0.52 (*SD* = 0.73); (*t*(86.39) = 1.518); *p* = 0.133; *d* = 0.21, 95% CI [−0.01, 0.44]). As expected, PLE-High were more confident in their final estimates at a medium to large effect size (Confidence PLEs-High: 2.84 (*SD* = 0.58), Confidence PLEs-Low: 2.41 (*SD* = 0.54); (*t*(89.167) = 6.475); *p* < 0.001; *d* = 0.80, 95% CI [0.57, 1.03]). Correlations between subjective competence and confidence were small and significant on trend level in PLEs-High [*r* = 0.206; *t*(78) = 1.857; *p* = 0.067], medium and significant in PLEs-Low [*r* = 0.342; *t*(1,104) = 12.089; *p* < 0.001], and medium and significant in the entire sample [*r* = 0.351; *t*(1,184) = 12.901; *p* < 0.001]. The moderation analysis with confidence as response variable, subjective competence as predictor and group as moderator revealed a significant model [*F*_(3,1,182)_ = 67.1; *p* < 0.001; *R*^2^_adjusted_ = 0.143]. Significant predictors in this model were subjective competence and group (both *p* < 0.001). The interaction term, however, was not significant (*p* = 0.298). This means, confidence in the estimates were mainly driven by subjective competence, neither proneness to PLEs nor an interaction of both. Of note, PLE-High reported higher subjective competence than PLE-Low with medium effect size (Subjective Competence PLEs-High: 3.85 (*SD* = 0.81), Subjective Competence PLEs-Low: 3.36 (*SD* = 0.83); (*t*(91.233) = 5.173); *p* < 0.001; *d* = 0.59, 95% CI [0.36, 0.82]). In sum, hypotheses 1 and 4 were supported, hypothesis 5 partially and hypotheses 2 and 3 not supported.

### Exploratory Analysis

As described in the Methods section, each trial followed a specific rationale. Hence, we continued with our exploratory analysis, presented trial wise. For this, we plotted per trial the estimates relative to the initial estimate in percentage. For example, if someone adjusted their estimates from an initial estimate of 30–33 following advice, the relative adjustment would be (33-30)/30 = 10%. This exploratory analysis follows the second aim of this study, which is to improve the JAS paradigm and to provide researchers with insights on different manipulation for future uses of the Aberrant-JAS.

#### Trial 1

**Figure 2:**
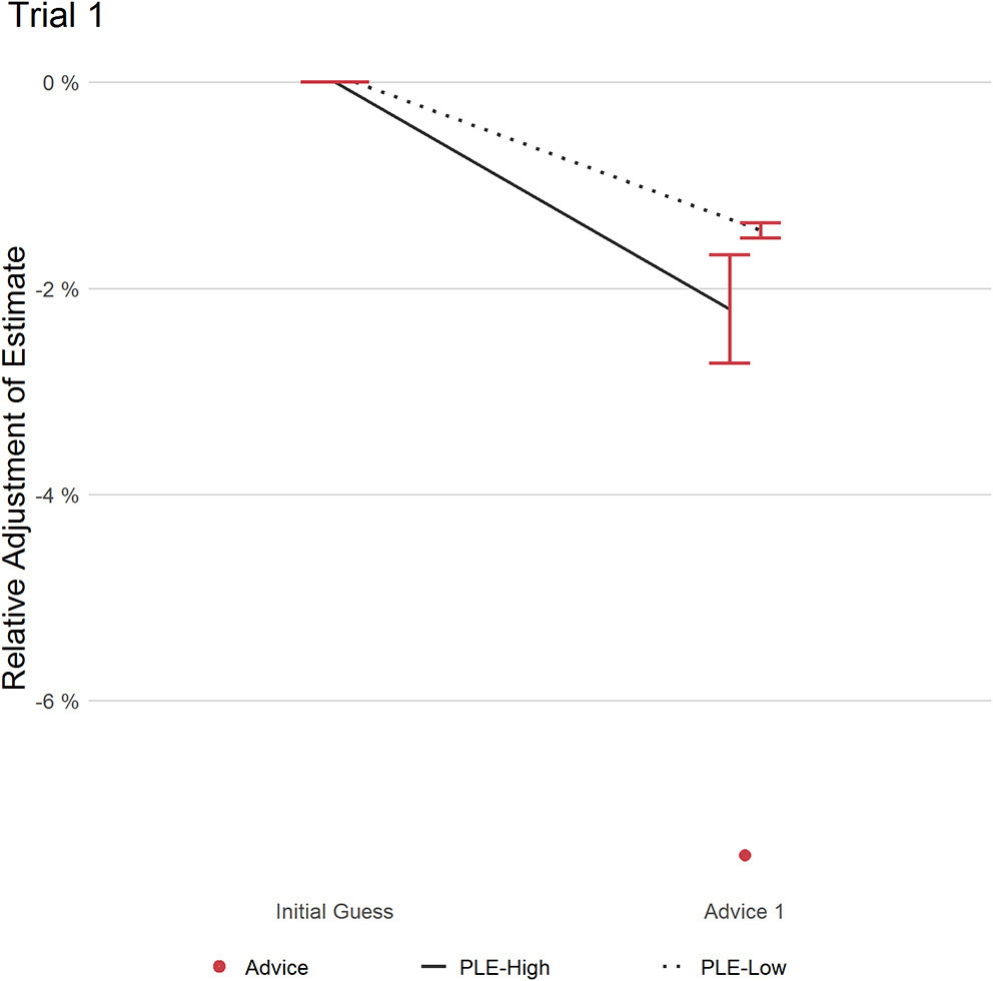
This figure displays the relative adjustment of estimates per group in trial 1. Red dot refers to the distance of advice relative to the initial estimate. Error bars refer to the standard error of the mean.

Descriptively observable in Figure 2, PLEs-High adjusted their estimate more than PLEs-Low in response to the advice, which did not reach significance (RAAW/RCAW PLEs-High: 0.29, RAAW/RCAW PLES-Low: 0.19; (*t*(82.457) = 1.480); *p* = 0.143; *d* = 0.28, 95% CI [0.06, 0.51]). As this trial consisted of one piece of advice only, RAAW and RCAW scores were identical.

#### Trial 2

**Figure 3:**
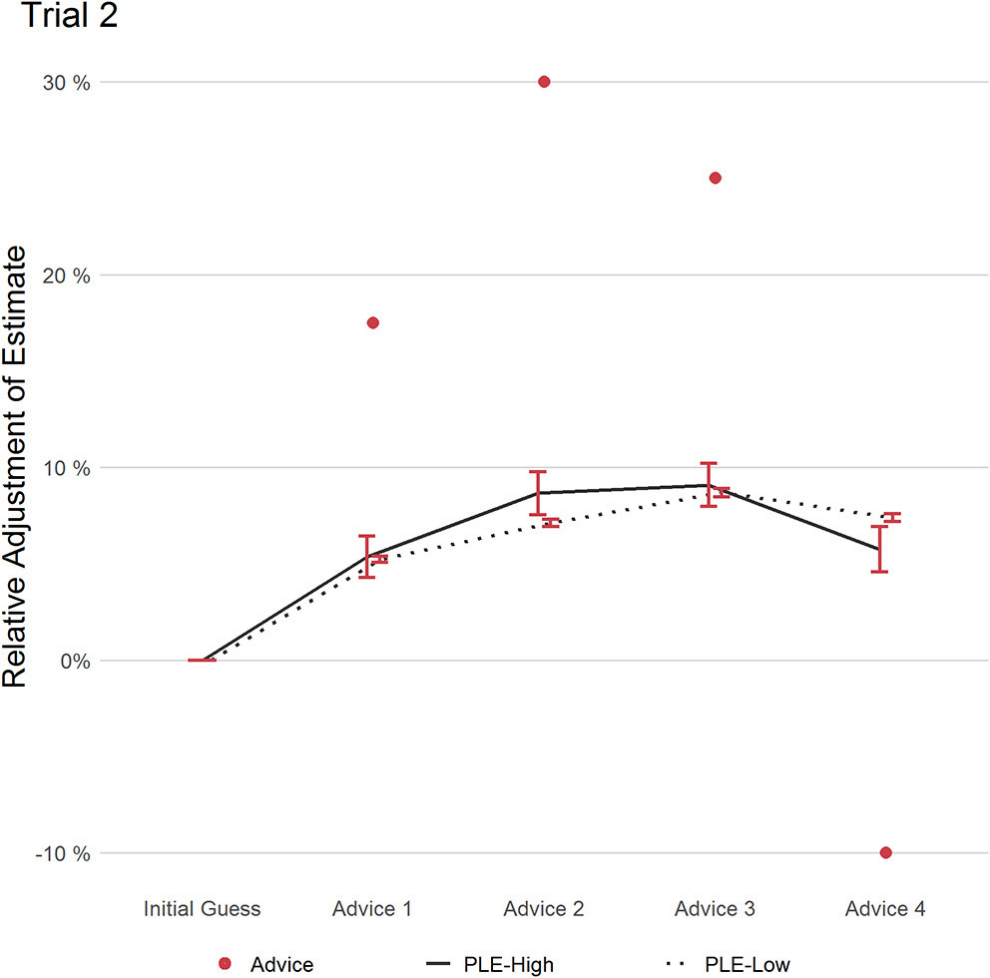
This figure displays the relative adjustment of estimates per group over all pieces of advice in trial 2. Red dots refer to the distance of each advice relative to the initial estimate. Error bars refer to the standard error of the mean.

Figure 3 shows the adjustment of the estimates relative to the initial estimate over different pieces of advice (depicted as red dots). Both groups adjusted similarly after the first advice. However, the second advice—deviating the most from the initial estimate—led to slightly stronger adjustment in the PLEs-High group as indicated by a higher RCAW score compared to PLEs-Low of medium effect size (PLEs-High: 0.31, PLEs-Low: 0.08; (*t*(79.233) = 1.788); *p* = 0.078; *d* = 0.68, 95% CI [0.45, 0.91]). PLEs-High also weighted advice 4—the outlier in this trial—more than PLEs-Low, even though this difference has also not reached significance (PLEs-High: 0.22, PLEs-Low: 0.05; (*t*(80.418) = 1.746); *p* = 0.085; *d* = 0.46, 95% CI [0.23, 0.69]). The increased advice weighting of PLEs-High in response to the outlier was driven by two factors: First, almost half of PLEs-High (47.5%) adjusted their estimate following the outlier, while only around one in four (28.2%) in the PLEs-Low group adjusted theirs (*t*(88.428) = 3.345; *p* = 0.001; *d* = 0.43, 95% CI [0.20, 0.65]). Second, of the participants who changed their estimate in response to the outlier, PLEs-High weighted the advice more strongly than PLEs-Low at a medium effect size, even though this difference did not reach significance in this subgroup analysis (PLEs-High: 0.46, PLEs-Low: 0.16; (*t*(38.907) = 1.461); *p* = 0.152; *d* = 0.44, 95% CI [0.10, 0.78]). In summary and in line with the theoretical assumptions, PLEs-High tended to weight the most extreme advice 2 and the outlier (advice 4) stronger than PLEs-Low.

#### Trial 3

**Figure 4:**
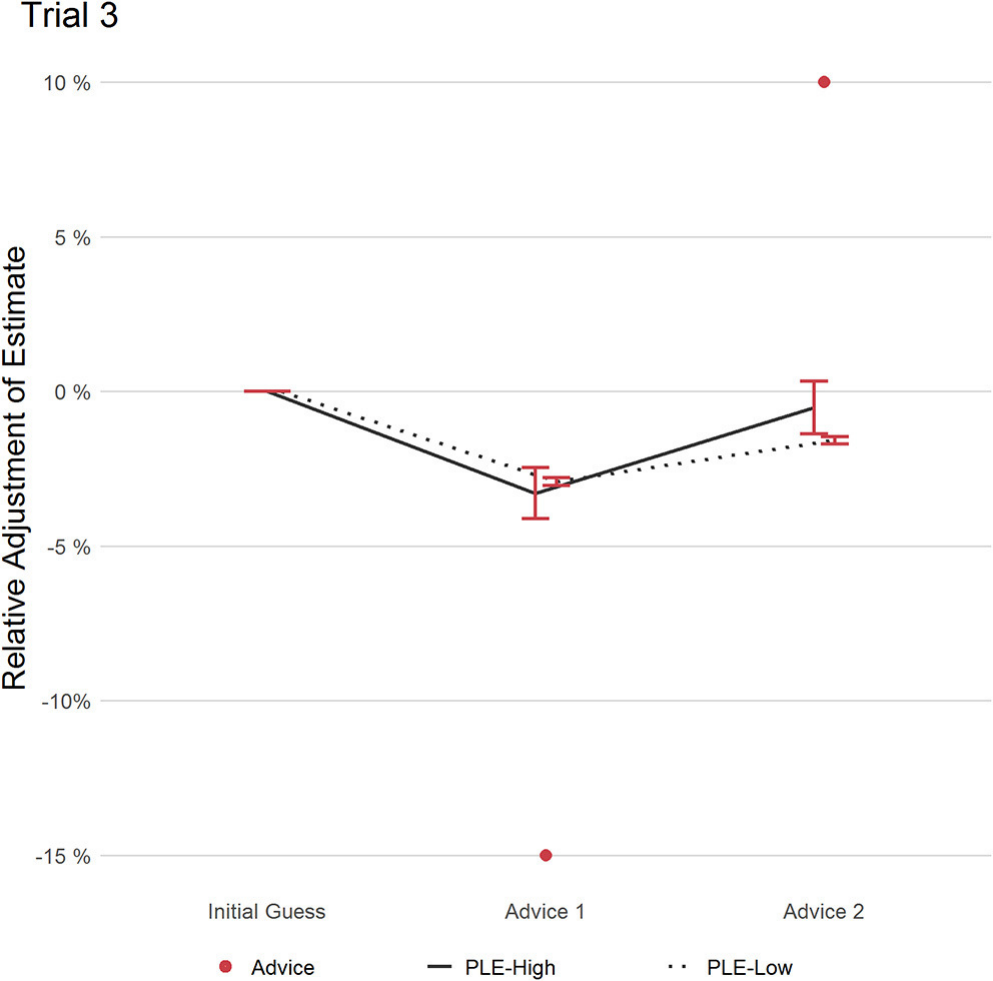
This figure displays the relative adjustment of estimates per group over all pieces of advice in trial 3. Red dots refer to the distance of each advice relative to the initial estimate. Error bars refer to the standard error of the mean.

In trial 3 (see Figure 4), participants saw two contrasting pieces of advice with the first advice being 15% lower and the second advice being 10% higher than the individual participant's initial estimate. After the first advice, both groups made similar adjustments of their estimate. The second advice, however, revealed a group difference in RCAW scores, which bordered significance (PLEs-High: 0.18, PLEs-Low: 0.08; (*t*(77.784) = 1.748); *p* = 0.084; *d* = 0.43, 95% CI [0.19, 0.66]). PLEs-Low were reluctant to change their estimate back to their first estimate in response to advice 2: Relative to the initial estimate, PLEs-Low adjusted their estimate by 1.6% which was closer to the averaged advice (2.5%) than their initial estimate. Hence, the resulting RAAW score was large for PLEs-Low (0.70). Cautiously, this could be interpreted as indicative that PLEs-Low was less ready to adjust their estimate in light of contradicting information despite the cost of ending up leaning to one side of advice.

#### Trial 4

**Figure 5:**
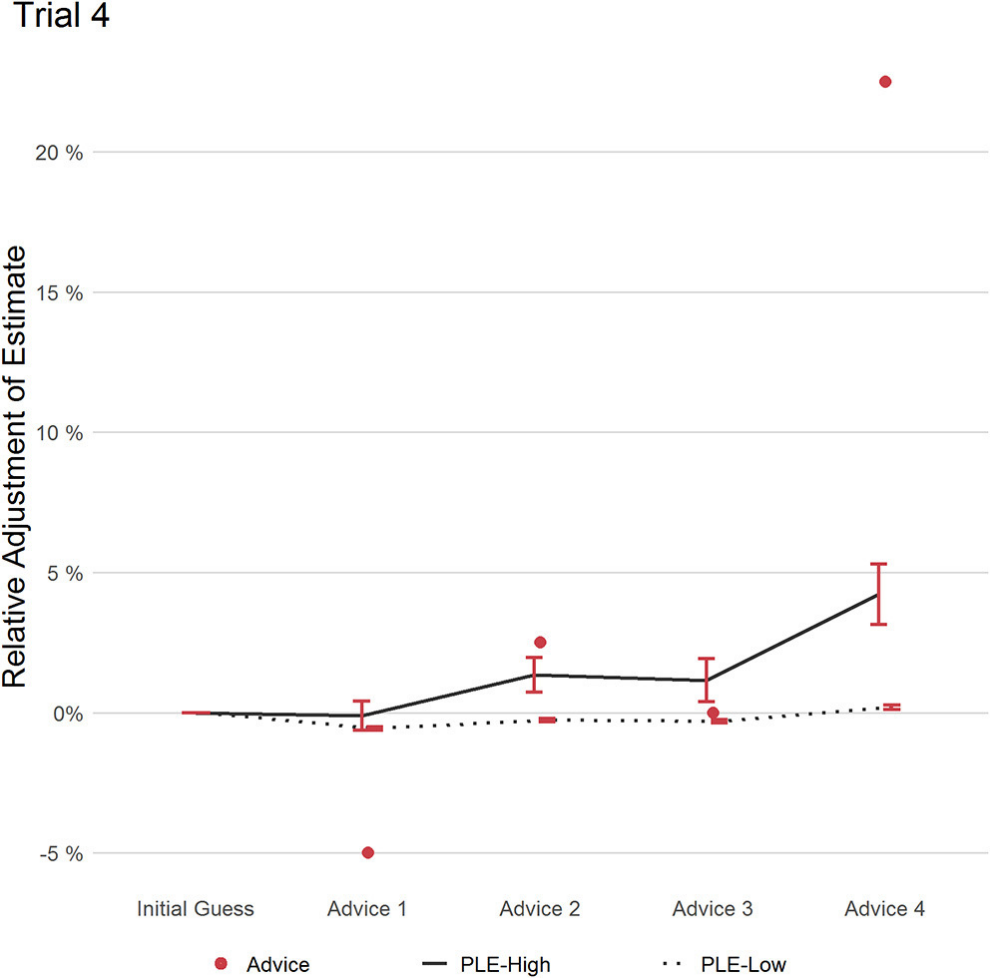
This figure displays the relative adjustment of estimates per group over all pieces of advice in trial 4. Red dots refer to the distance of each advice relative to the initial estimate. Error bars refer to the standard error of the mean.

Trial four, see Figure 5, consisted of four pieces of advice. The first three deviated only little from the initial estimate, therefore both groups showed only little deviation from their initial estimate. Advice four deviated from the first three pieces of advice. While PLEs-Low weighted this last piece of advice only marginally (RCAW = 0.02), PLEs-High weighted it heavily (RCAW = 0.18). This difference in RCAW scores on the fourth advice was significant with a large effect size (*t*(79.746) = 3.398; *p* = 0.001, *d* = 1.07, 95% CI [0.84, 1.30]), and as a result also the weighting of the advice averaged in this trial RAAW, again with a large effect size (PLEs-High: 0.81; PLEs-Low: 0.03; (*t*(80.016) = 3.807); *p* < 0.001; *d* = 1.10, 95% CI [0.87, 1.34]). Similar to trial 2, more participants of the group PLEs-High adjusted their estimate (36.31%) in response to the outlier of advice 4, compared to participants in group PLEs-Low (12.61%), revealing a significant medium effect (*t*(84.465) = 4.304); *p* < 0.001; *d* = 0.69, 95% CI [0.46; 0.92]). Furthermore, of all participants who adjusted their estimate, PLEs-High weighted the last piece of advice more strongly (RCAW PLEs-High: 0.50; RCAW PLEs-Low: 0.15; (*t*(30.596) = 3.150); *p* = 0.003; *d* = 1.01, 95% CI [0.59; 1.43]). To conclude, even after three pieces of advice deviating from the initial estimate only marginally, thereby “confirming” the initial estimate, PLEs-High weighted the fourth advice, deviating from the initial estimate and the previous advice strongly, more than PLEs-Low.

#### Trial 5

**Figure 6:**
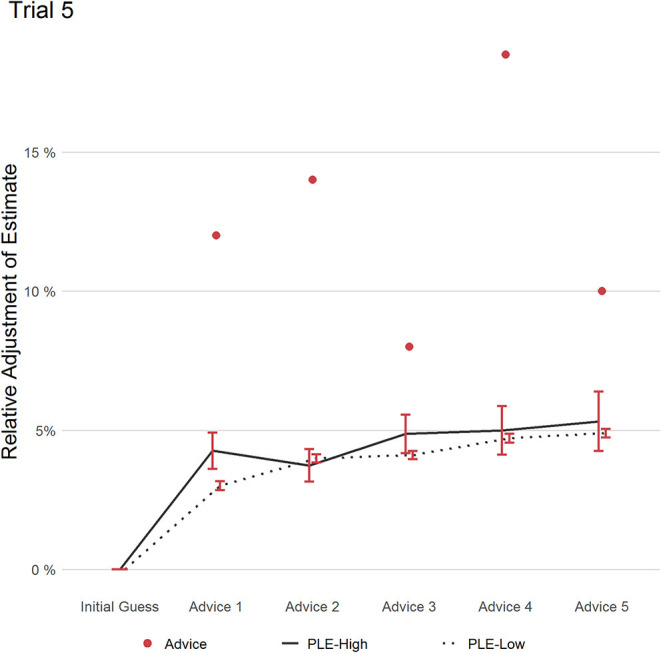
This figure displays the relative adjustment of estimates per group over all pieces of advice in trial 5. Red dots refer to the distance of each advice relative to the initial estimate. Error bars refer to the standard error of the mean.

In trial 5 (see Figure 6), participants saw five pieces of advice, all hinting in the same direction by similar magnitude. PLEs-High had at trend-level slightly higher RCAW scores in response to advice 1 (PLEs-High: 0.35, PLEs-Low: 0.25; (*t*(89.936) = 1.746); *p* = 0.084; *d* = 0.21, 95% CI [−0.02, 0.44]). From advice 2 to 5, both groups paralleled mostly in their advice weighting with no significant differences in RCAW scores (*p* ≥.105). However, RCAW scores averaged over the five pieces of advice revealed a borderline-significant group difference with medium effect size (PLEs-High: 0.23, PLEs-Low: 0.11; (*t*(80.07) = 1.797); *p* = 0.076; *d* = 0.51, 95% CI [0.29, 0.74]). Still after five pieces of advice, both groups have adjusted their estimates similarly according to RAAW scores (PLEs-High: 0.43, PLEs-Low: 0.39; (*t*(82.323) = 0.412); *p* = 0.681; *d* = 0.08, 95% CI [−0.14; 0.31]). That means, multiple uniform pieces of advice did not lead to stronger adjustment by any group.

### Advice Weighting and Self-Esteem

In exploratory fashion, we investigated the relationship between self-esteem and performance in the JAS-task. Looking at both the entire sample and the group PLEs-High, self-esteem did neither correlate with RCAW [entire sample: *r* = −0.005; (*t*(1,184) = 0.161); *p* = 0.872; PLEs-High: *r* = 0.071; (*t*(78) = 0.627); *p* = 0.532] nor RAAW [entire sample: *r* = −0.035; (*t*(1,184) = 1.203); *p* = 0.229; PLEs-High: *r* = −0.107; (*t*(78) = 0.953); *p* = 0.344] or NoPA [*r* = −0.038; (*t*(1,184) = 1.314); *p* = 0.189; PLEs-High: *r* = 0.019; (*t*(78) = 0.170); *p* = 0.866]. However, higher scores in global self-esteem were related to higher confidence in their estimate in the entire sample [*r* = 0.171; (*t*(1,184) = 5.955); *p* < 0.001]. Within PLEs-High, the correlation was the same (*r* = 0.178) but did not reach significance [*t*(78) = 1.601; *p* = 0.114].

### Task Evaluation

After the task, we asked participants to rate five statements pertaining to the task, revealing two group differences regarding the task: PLEs-High found the correct answer less important and more readily believed that advice was “just there to mislead.” For exact wording and statistics, see Table 3.

**Table 3:** Endorsements toward statements of the task.

	**PLEs-High**	**PLEs-Low**	**Group differences (Welch's** ***t*****-test)**	
	**(*n* = 80)**	**(*n* = 1,106)**		
The correct answer was not important to me.	2.71 (*SD =* 1.26)	2.28 (*SD =* 1.10)	*t*(87.845) = 2.982	*p =* 0.004
I wanted to trust my first impression.	3.95 (*SD =* 1.01)	4.08 (*SD =* 0.74)	*t*(85.346) = 1.147	*p =* 0.255
The task was fun.	3.96 (*SD =* 1.25)	4.01 (*SD =* 0.92)	*t*(85.349) = 0.366	*p =* 0.716
The previous answers by other participants were just there to mislead me.	3.51 (*SD =* 1.03)	2.93 (*SD =* 1.00)	*t*(90.036) = 4.910	*p <* 0.001
It was annoying to see more previous answers by other participants than I wanted to.	3.58 (*SD =* 1.23)	3.49 (*SD =* 1.31)	*t*(92.36) = 0.581	*p =* 0.563

*Scaled from 1 “strongly disagree” to 5 “strongly agree.”; PLEs, psychotic-like experiences*.

## Discussion

### Summary of Main Findings

This study aimed to investigate how people with more frequent psychotic-like experiences (PLEs) integrate information using an adapted Judge-Advisor System (JAS), the *Aberrant JAS*. Participants estimated a person's age and could change their estimate in response to consecutively provided advice in the form of (fabricated) answers by previous participants. The degree to which a participant adjusted their estimates gives a clear measure as to how much this participant weighted the newly presented information.

We expected participants with more frequent PLEs to weight the currently available advice more than participants with less frequent PLEs. This preregistered hypothesis was supported. However, adjustments at the end of each trial did not differ between both groups, as indicated by advice weighting scores considering all pieces of advice averaged per trial. Unexpectedly, participants with more frequent PLEs preferred to see more advice than people with less frequent PLEs. In this regard this study adds to the increasing literature failing to replicate the jumping to conclusions account on some paradigms (38–41). Yet, people with more frequent PLEs were more confident in their final estimate compared to participants with less frequent PLEs. We did expect an increased confidence due to the same finding in the forerunner study (13) and previous findings on overconfidence related to psychotic experience (5). However, overconfidence usually refers to false answers. This group difference using this somewhat difficult task to estimate one's age purely from a photograph thus adds to the literature on overconfidence. Confidence in one's response was predicted by subjective competence in estimating ages rated before the task. However, this relation was similar in both groups and there was no moderation by group (and thus there is no indication that the link between subjective competence and confidence is somehow different in people with more frequent PLEs). In sum, hypotheses 1 and 4 were supported, hypothesis 5 was partially and hypotheses 2 and 3 were not supported.

In addition to the increased relative current advice weighting scores, *post-hoc* trial-wise analysis provided additional evidence that participants with more frequent PLEs put more weight toward currently available information than people with less frequent PLEs: In trial 4, the first three pieces of advice deviated only marginally from the initial estimate, with a fourth advice deviating largely. People with more frequent PLEs adjusted their estimate more often and more strongly in response to this new information than participants with less frequent PLEs. A similar pattern could be observed in trial 2, in which participants with more frequent PLEs did weight the fourth advice—an outlier as it hinted in the opposite direction to the initial estimate than the previous three pieces of advice (see Table 1)—more than participants with less frequent PLEs. In summary, people with more frequent PLEs, compared to people with less frequent PLEs, more readily accepted and integrated newly available (deviant) information while somewhat considering previous advice/information less.

### Increased Information Integration Explained by Hypersalience of Evidence-Matching Hypothesis, Unstable-Attractor Network, Circular Inference, and Liberal Acceptance

Results show an increased integration of currently available information by participants with more frequent PLEs, which thus supports the hypersalience of evidence-matching hypothesis model (18). The hypersalience of evidence-matching hypothesis model posits that patients with schizophrenia perceive new evidence that fits to a hypothesis as “hypersaliently fitting”, leading them to increase their conviction in this hypothesis, while they give the same evidence less weight for a re-evaluation of the contrary hypothesis.

To illustrate how this model translates to participants' behavior in our study, especially the strong correction of the estimate in response to the outlier advice in trial 2 and 4 by participants with more frequent PLEs: The (fabricated) advice suggesting that the person on the photo may be older than previously thought “hypersaliently” points toward the idea (hypothesis) that the person is older than originally thought. This hypersalience might then also lead to an ignoring of one's own initial estimate as well as previous advice.

This neglect of previous advice, once new advice is presented could also be explained by the unstable-attractor network (42), according to which patients with schizophrenia have an increased instability in cognition. In their analysis of the beads task (variant of the fish task), Adams et al. (42) showed that patients with schizophrenia updated their probability estimates more in response to unexpected input and less to consistent input. In a design related to the original fish task, Jardri et al. (43) investigated the certainty toward either lake after the first catch, given a prior probability for either lake to be chosen. The authors could show an “under-weighting of priors” and explain this with the “circular inference” stemming from an excitatory/inhibitory imbalance in hierarchical neural processing: Ascending inference loops—a top-down approach leading to interpret current sensory information as prior knowledge (“expect what we see”)—are stronger in schizophrenia patients than in controls. These ascending inference loops could explain that participants with more frequent PLEs under-weight previous advice once new advice is presented; as could be observed by the increased weighting of outliers in trials 2 and 4.

An alternative explanation for the increased weighting of current information by participants with more frequent PLEs in our JAS paradigm is liberal acceptance (2, 44, 45): People with a liberal acceptance bias (which is assumed to be more present in people with PLEs and schizophrenia) have a decreased decision threshold for accepting a hypothesis. For example, individuals with schizophrenia put an increased likelihood-rating to conclusions that controls judge as unlikely (46). Similarly, individuals with schizophrenia decide for a lake on the fish task at a lower probability rating (47). Likewise, “liberally accepting” another person's estimate as likely/correct could explain why people with more frequent PLEs put more weight to advice from unknown “previous participants.”

### Increased Information Integration and Belief Inflexibility

However, other findings suggest individuals with schizophrenia show a decreased integration of new information and a general belief inflexibility [for a review, see Eisenacher and Zink (4)]. For example in the bias against disconfirmatory evidence paradigm using delusion-neutral material (15) psychosis-prone individuals correct the likelihood rating of scenarios disconfirmed in light of new information to a lesser degree than controls (16, 48). This task behavior has been linked to a lack of evidence integration (integration of disambiguating information) rather than conservatism (unwillingness to give high likelihood ratings) (49, 50). Belief inflexibility in individuals with schizophrenia was also shown in the *What is this?* Task by Serrano-Guerrero et al. (51). Further, from a clinical perspective, individuals with delusions show a strong belief inflexibility regarding their delusions (14, 52).

Thus, one should be particularly careful concluding from this study's results that individuals with more frequent PLEs are generally more ready to change beliefs in light of new information. Instead, future studies should clarify under which conditions psychosis-prone individuals accept new input for the formation of beliefs and under which conditions beliefs are upheld despite disconfirmatory input. These somehow contradictory processes have recently been integrated in two independent reviews by Ward and Garety (53) and Moritz et al. (2) in which one process related to the formation of delusional beliefs and the other to its maintenance.

Furthermore, in a related probabilistic advice-taking task, participants with more frequent PLEs did use less advice and assumed advice to be more intentionally misleading than participants with less frequent PLEs (54). However, there is an important difference to the Aberrant JAS we use: Participants in Wellstein's task had to choose between two colors, for which they had to rely either on a partly volatile advisor or on a non-social cue as they had no additional information on which color to choose. Whereas, participants in our task could also rely on their own judgement and were thus less dependent on advice.

### The Novelty of the Aberrant JAS

We want to point out two important aspects in which the Aberrant JAS differs from classical tasks capturing reasoning in relation to psychosis. Compared to the beads or fish task—from which relevant models (hypersalience of evidence-matching hypothesis, and unstable-attractor network, circular inference) are derived—the Aberrant JAS is not a probabilistic reasoning task, where an optimal solution can be derived. Estimating someone's age based on a photograph “correctly” is very difficult and there is also no obvious optimal solution to advice weighting, especially with multiple contradicting advice (37, 55). At the same time, the Aberrant JAS is presumably easier to understand and involves a scenario much more likely to encounter in the real world. It also provides a much more direct measure on how information is being integrated in a judgement and does not rely on probability or confidence ratings from a rather artificial reasoning task as a measure for information weighting.

Further, the Aberrant JAS is a social task. While social frameworks are a frequent contextual factor for the exacerbation of positive symptoms, the social nature of the task adds noise as different groups possibly have different assumptions about advice (e.g., assuming advice to be hostile). On the other hand, belief formation is a social process (56). Thus, we can assume a special importance of social processes in belief formation in schizophrenia (53). For example, Jolley et al. (57) found that patients with caregivers show much increased belief flexibility than those with no caregivers. This highlights the importance to investigate belief flexibility and cognitive biases in social contexts.

### Limitations and Outlook

This study has important limitations. For example, we have investigated a community sample with no further information on the clinical status of participants. Further, our sample has also shown differences in demographic variables (e.g., age, education). Performance on cognitive bias paradigms have shown to be affected by pharmacological treatment (28, 29), psychological interventions (58), need for care (59), current symptomology (16), and stress (60). Thus, this study needs to be replicated within clinical samples under consideration of these possible confounds. Future studies should also validate the task further, for example by comparing task performance to the bias against disconfirmatory evidence paradigm, by investigating different estimation tasks, or by providing more background information about the advisors. While we believe our differently designed trials (e.g., in terms of number and distance of advice) provide valuable insights for other researchers to design their trials, this variance in trial design likely decreased power. Thus, future studies may focus on specific trial design, for example on trials with outliers only. Finally, *p*-values in exploratory trial-wise analysis need to be treated with caution (61). While all exploratory analyses were based on theoretical assumptions, they were mainly data-driven generating an ignored alpha-error accumulation through multiple *post-hoc* tests. However, our main analyses were preregistered, an important corner-stone in good scientific practice (62).

## Conclusion

This study introduced the intuitive Aberrant JAS, an adapted JAS-paradigm. The Aberrant JAS captures—within a social framework—information integration relevant to cognitive biases related to psychosis. As expected, participants with more frequent PLEs adjusted their estimates more readily toward currently available new advice, especially if this advice was an outlier (differing from previous pieces of advice and one's initial estimate). This increased readiness of participants with more frequent PLEs to change their estimate due to new incoming information challenges previous accounts on a general inflexibility in revising conclusions, but supports models predicting an “overcorrection” due to an elevated weighting of incoming information, which have been related to the formation and maintenance of psychotic experiences or delusions, for example in schizophrenia.

## Data Availability Statement

The datasets presented in this article are not readily available because datasets are only available upon legitimate requests. Requests to access the datasets should be directed to JS, j.scheunemann@uke.de.

## Ethics Statement

The studies involving human participants were reviewed and approved by Lokale Psychologische Ethikkommission (LPEK) am Zentrum für Psychosoziale Medizin—Martinistrasse 52 20246 Hamburg Germany. The patients/participants provided their written informed consent to participate in this study.

## Author Contributions

JS, RF, and SM developed the research idea. SM was PI of the study. RF contributed largely to the discussion. All authors have written and proof-read the manuscript.

## Conflict of Interest

The authors declare that the research was conducted in the absence of any commercial or financial relationships that could be construed as a potential conflict of interest.

## Footnotes

^1^*As one of the reviewers pointed out, we should have debriefed participants at the end of the experiment that advice was fabricated*.
