# Supplementary material for: Probing the Hypersalience Hypothesis—An Adapted Judge-Advisor System Tested in Individuals With Psychotic-Like Experiences
Source: Front Psychiatry. 2021 Mar 4;12:612810. doi: 10.3389/fpsyt.2021.612810 (PMC7969715; doi:10.3389/fpsyt.2021.612810)
Supplement: Supplementary file 1 [file Table_1.pdf]

Supplementary Table 1

Self-reported Lifetime Diagnoses

	PLEs-High (n = 80)	PLEs-Low (n = 1,106)
No psychiatric diagnosis	48.8%	59.0%
Schizophrenia/psychosis	0%	0.4%
Depression	33.8%	26.1%
Anxiety disorder	37.5%	25.6%
Posttraumatic stress disorder	11.2%	5.4%
Obsessive-compulsive disorder	5.0%	2.5%
Bipolar disorder	5.0%	3.5%
Eating disorder	7.5%	2.1%
Substance or alcohol dependence	3.8%	3.7%
Personality disorder	2.5%	1.7%
Attention Deficit Hyperactivity Disorder	6.2%	3.5%
Autism Spectrum Disorder	2.5%	0.7%
Other	1.2%	3.1%

Note. Multiple answers were possible. Group difference χ^2 (12) = 20.570, p = .057.
