# Supplementary material for: Probing the Hypersalience Hypothesis—An Adapted Judge-Advisor System Tested in Individuals With Psychotic-Like Experiences
Source: Front Psychiatry. 2021 Mar 4;12:612810. doi: 10.3389/fpsyt.2021.612810 (PMC7969715; doi:10.3389/fpsyt.2021.612810)
Supplement: Supplementary file 2 [file Data_Sheet_1.PDF]

ASPREDICTED

Testing Overcorrection in People with Psychotic-Like Experiences with the (#21768)

Author(s)
Jakob Scheunemann (University Medical Center Hamburg-Eppendorf) - j.scheunemann@uke.de
Rabea Fischer (University Medical Center Hamburg-Eppendorf) - r.fischer@uke.de
Steffen Moritz (University Medical Center Hamburg-Eppendorf) - moritz@uke.de

Created: 04/05/2019 05:24 AM (PT)
Public: 04/07/2019 01:32 PM (PT)

1) Have any data been collected for this study already?
No, no data have been collected for this study yet.

2) What's the main question being asked or hypothesis being tested in this study?
We propose that in a judge-advisor system, people with elevated psychotic-like experiences (compared to people with low levels of psychotic-like experiences) will (a) weight the current advice more, (b) weight average advice more, (c) prefer less advice before making a final judgement, and will (d) be more confident in their final judgement. Further, we assume (e) that confidence correlates positively with subjective competence, moderated by group.

3) Describe the key dependent variable(s) specifying how they will be measured.
The dependent variables are (a) relative current advice weighting ([new estimate – previous estimate]/[current advice-previous estimate]), (b) relative average advice weighting ([final estimate – initial estimate]/[mean advice-initial estimate] (c) the number of times the participant preferred to see additional advice after each presented advice (simulated decision), (d) the confidence rating after the final judgement (confidence scale 1-4), and (e) subjective competence ("how good do you judge yourself to be at estimating other people's age?") on a 5-point scale, asked before the task. Variables A-D will be averaged on a subject level across all five trials, and variable A additionally across multiple estimates within each trial.

4) How many and which conditions will participants be assigned to?
Based on the subscale positive symptoms of the Community Assessment of Psychotic Experiences (CAPE), participants will be divided into two groups: PLEs-High (at least 2SD above the mean), PLEs-Low (maximum 0.5 SD above the mean). All participants receive the same conditions in the same order, only the order of picture stimuli is randomized.

5) Specify exactly which analyses you will conduct to examine the main question/hypothesis.
We plan to calculate Welch's t-tests to determine mean group differences for (a) relative current advice weighting, (b) relative average advice weighting, (c) preference to see more advice, and (d) confidence. We will (e) calculate a Pearson's correlation between subjective competence and confidence for both groups. Trial 2 and 4 each contain one outlier in the advice given, which, if hypothesis A holds true, should lead to increased relative current advice weighting scores in response to those outliers.

6) Describe exactly how outliers will be defined and handled, and your precise rule(s) for excluding observations.
Participants will be excluded if they failed either the explicit attention test (rating ≤5 on a 7-point scale of attention during study), the implicit attention test (an item within the socio-demographic questionnaire at the end of the survey), or if they showed excessive speeding (taking less than 50% of the median completion time for the complete study).

7) How many observations will be collected or what will determine sample size? No need to justify decision, but be precise about exactly how the number will be determined.
Based on budget, the predetermined number of participants is 1,500.

8) Anything else you would like to pre-register? (e.g., secondary analyses, variables collected for exploratory purposes, unusual analyses planned?)
We will also measure self-esteem via the Rosenberg-self-esteem scale, which we will analyze exploratorily. Additionally, we will ask participants to rate their subjective competence after the task, the same item as for variable E and also ask participants to compare their subjective competence to others (same as variable E, but with choices "above average", "average", and "below average") before the task. After the task, participants are asked to respond to five statements about the task. We will recruit participants via Amazon Mechanical Turk, where we will predetermine to include only participants with an IP address from the United States and an approval rate of at least 95 percent based upon at least 100 previous tasks, and exclude participants, who had participated in previous studies by our working group.
